# Draft genome sequence of *Limosilactobacillus fermentum* isolated from subgingival biofilm

**DOI:** 10.1128/mra.00280-23

**Published:** 2024-02-05

**Authors:** Eun-Young Jang, Seok Bin Yang, Jeewan Chun, Cheul Kim, Ji-Hoi Moon, Jae-Hyung Lee

**Affiliations:** 1 Department of Oral Microbiology, College of Dentistry, Kyung Hee University, Seoul, South Korea; 2 Department of Dentistry, Graduate School, Kyung Hee University, Seoul, South Korea; 3 Research Institute of Oral Science, College of Dentistry, Gangneung-Wonju National University, Gangneung, South Korea; University of Maryland School of Medicine, Baltimore, Maryland, USA

**Keywords:** *Limosilactobacillus fermentum*, genome, subgingival biofilm

## Abstract

*Limosilactobacillus fermentum* is generally considered beneficial for vaginal and digestive health. However, strains isolated from the oral cavity, especially from periodontitis lesions, have not been thoroughly studied. Here, we report the draft genome sequence of strain KHUD_007 isolated from the subgingival biofilm of a Korean patient with periodontitis.

## ANNOUNCEMENT

*Limosilactobacillus fermentum* is a Gram-positive, non-spore-forming rod-shaped bacterium (1). While some strains from food have beneficial effects (2, 3), not all *L. fermentum* strains are probiotic. Some strains have been associated with diseases, such as dental caries (4, 5) and colon inflammation (1), which suggest that *L. fermentum*, typically considered mutualistic, can become pathogenic due to factors such as habitat, sub-species variation, and host interactions (6). Out of the 183 *L*. *fermentum* genomes in GenBank, the majority are derived from strains isolated from food or feces. There are fewer than 15 genomes of oral-derived strains, and none are derived from periodontitis lesions.

This study performed genome sequencing of strain KHUD_007 to address the lack of studies on oral-derived *L. fermentum*. The strain was obtained from the subgingival biofilm of a Korean patient with periodontitis in 2016, approved by the Institutional Review Board (IRB) at the Dental Hospital of Kyung Hee University (KHD IRB 1606-5). Before genome sequencing, we obtained the 16S rRNA gene sequence of KHUD_007 using universal eubacteria primers (Table 1) (7). The obtained sequence was then compared using the standard BLAST against the nucleotide collection database with default parameters for bacterial species identification. For genome sequencing, KHUD_007 was anaerobically cultured in Difco Lactobacilli MRS broth at 37°C overnight. The genomic DNA extracted utilizing GenElute Bacterial Genomic DNA Kits (Sigma-Aldrich) was used for library construction using TruSeq DNA PCR-Free kit. Briefly, genomic DNA was mechanically sheared to 400–500 bp, and the resulting overhangs were converted to blunt ends. After a cleaning step using the AMPure XP Beads, the 3′ ends of the fragmented genomic DNA were first adenylated and then ligated to the index adapters. DNA fragments containing adapter sequences (500–600 bp) were extracted from a 2% agarose gel and then enhanced via PCR using adapter-specific primers. The resulting library was sequenced in paired-end mode on the Illumina Hi-Seq X platform. Default parameters were used for all bioinformatic analysis tools unless otherwise specified. We obtained 9,635,801 pairs of raw sequencing reads (2 × 151 bp). All the high-quality reads were used for genome assembly after assessing the quality of the raw sequencing reads using FASTQC (v0.12.1) (https://www.bioinformatics.babraham.ac.uk/projects/fastqc/). The adapter sequences in the raw reads were trimmed using cutadapt software (v1.18) with “-a AGATCGGAAGAGC-A AGATCGGAAGAGC -m 1” parameters (8). We constructed the genome of *L. fermentum* KHUD_007 using SPAdes (v3.15.3) (9). The NCBI Prokaryotic Genome Annotation Pipeline (v6.2) (10) was used for gene annotation of the genome.

**TABLE 1:** Information of 16S rRNA gene and genome assembly of *Limosilactobacillus fermentum* KHUD_007

16S rRNA gene sequencing	
GenBank accession	PP106247
Forword primer (5′–3′)	AGAGTTTGATCCTGGCTCAG
Reverse primer (5′–3′)	GGTTACCTTGTTACGACTT
Reaction mixture (20 µL)	0.5 µg DNA template, 200 µM each primer, 250 µM each dNTPs, and AccuPower HotStart Pfu PCR PreMix (Cat. K-2301, Bioneer, Korea)
Amplification condition	95°C 5 min and 30 cycles of 95°C 20 s, 53°C 20 s, and 72°C 60 s
Sequencing kit and system	BigDye Terminator v3.1 and ABI3730XL (Applied Biosystems, USA)

Genome assembly feature	
GenBank accession	JANFGS000000000.1
Assembly statistics	
Total length (bp)	1,993,541
No. of contigs (≥500 bp)	124
N50 (bp)	44,326
L50 (contig)	12
GC content (%)	52.21
Genome coverage	1411.0×
Genome features	
Total genes	2,045
Protein-coding genes	1,910
tRNAs	54
Complete rRNAs (5S)	1
Partial rRNAs (16S, 23S)	2, 3
ncRNAs	3
Pseudogenes	72

*L. fermentum* KHUD_007 genome is 1,993,541 bp with G + C content of 52.21% (Table 1). A total of 124 contigs (≥500 bp), 1,910 protein-coding genes, 6 rRNAs, and 54 tRNAs were identified. The genome coverage is 1411.0×, and the contig N50 value for the genome is 44,326. The genome completeness is 92.76% based on CheckM analysis (v1.2.2) (11). The genomic information of *L. fermentum* KHUD_007 presented here can be utilized in future studies to investigate the subspecies-level variation of *L. lactobacillus* in various habitats, including the oral cavity, and its potential relationship with diseases.

## Data Availability

The draft genome sequence was deposited at DDBJ/ENA/GenBank under the accession JANFGS000000000.1 via BioProject PRJNA860016 (with BioSample accession SAMN29815206, SRA accession SRX20733841, and GenBank assembly accession GCA_024385625.1).

## References

[1] Bailie MA, Altermann E, Young W, Roy NC, McNabb WC. 2021. Complete annotated genome sequence of Limosilactobacillus fermentum AGR1487. Microbiol Resour Announc 10:e01056-20. doi:10.1128/MRA.01056-20 33414290 PMC8407690

[2] Kaewnopparat S, Dangmanee N, Kaewnopparat N, Srichana T, Chulasiri M, Settharaksa S. 2013. In vitro probiotic properties of Lactobacillus fermentum SK5 isolated from vagina of a healthy woman. Anaerobe 22:6–13. doi:10.1016/j.anaerobe.2013.04.009 23624069

[3] Lacerda DC, Trindade da Costa PC, Pontes PB, Carneiro Dos Santos LA, Cruz Neto JPR, Silva Luis CC, de Sousa Brito VP, de Brito Alves JL. 2022. Potential role of Limosilactobacillus fermentum as a probiotic with anti-diabetic properties: a review. World J Diabetes 13:717–728. doi:10.4239/wjd.v13.i9.717 36188141 PMC9521441

[4] Badet C, Thebaud NB. 2008. Ecology of lactobacilli in the oral cavity: a review of literature. Open Microbiol J 2:38–48. doi:10.2174/1874285800802010038 19088910 PMC2593047

[5] Caufield PW, Schön CN, Saraithong P, Li Y, Argimón S. 2015. Oral lactobacilli and dental caries: a model for niche adaptation in humans. J Dent Res 94:110S–8S. doi:10.1177/0022034515576052 25758458 PMC4547204

[6] Eberl G. 2010. A new vision of immunity: homeostasis of the superorganism. Mucosal Immunol 3:450–460. doi:10.1038/mi.2010.20 20445502

[7] Srinivasan R, Karaoz U, Volegova M, MacKichan J, Kato-Maeda M, Miller S, Nadarajan R, Brodie EL, Lynch SV. 2015. Use of 16S rRNA gene for identification of a broad range of clinically relevant bacterial pathogens. PLoS One 10:e0117617. doi:10.1371/journal.pone.0117617 25658760 PMC4319838

[8] Martin M. 2011. Cutadapt removes adapter sequences from high-throughput sequencing reads. EMBnet J 17:10. doi:10.14806/ej.17.1.200

[9] Bankevich A, Nurk S, Antipov D, Gurevich AA, Dvorkin M, Kulikov AS, Lesin VM, Nikolenko SI, Pham S, Prjibelski AD, Pyshkin AV, Sirotkin AV, Vyahhi N, Tesler G, Alekseyev MA, Pevzner PA. 2012. SPAdes: a new genome assembly algorithm and its applications to single-cell sequencing. J Comput Biol 19:455–477. doi:10.1089/cmb.2012.0021 22506599 PMC3342519

[10] Tatusova T, DiCuccio M, Badretdin A, Chetvernin V, Nawrocki EP, Zaslavsky L, Lomsadze A, Pruitt KD, Borodovsky M, Ostell J. 2016. NCBI prokaryotic genome annotation pipeline. Nucleic Acids Res 44:6614–6624. doi:10.1093/nar/gkw569 27342282 PMC5001611

[11] Parks DH, Imelfort M, Skennerton CT, Hugenholtz P, Tyson GW. 2015. CheckM: assessing the quality of microbial genomes recovered from isolates, single cells, and metagenomes. Genome Res 25:1043–1055. doi:10.1101/gr.186072.114 25977477 PMC4484387

